# Conflict Test Battery for Studying the Act of Facing Threats in Pursuit of Rewards

**DOI:** 10.3389/fnins.2021.645769

**Published:** 2021-05-04

**Authors:** Elizabeth Illescas-Huerta, Leticia Ramirez-Lugo, Rodrigo O. Sierra, Jorge A. Quillfeldt, Francisco Sotres-Bayon

**Affiliations:** ^1^Cell Physiology Institute-Neuroscience, National Autonomous University of Mexico, Mexico City, Mexico; ^2^Department of Physiology, University of Szeged, Szeged, Hungary; ^3^Department of Biophysics, Biosciences Institute, Federal University of Rio Grande do Sul, Porto Alegre, Brazil

**Keywords:** approach-avoidance, choice, decision-making, prefrontal, amygdala, accumbens, fear

## Abstract

Survival depends on the ability of animals to avoid threats and approach rewards. Traditionally, these two opposing motivational systems have been studied separately. In nature, however, they regularly compete for the control of behavior. When threat- and reward-eliciting stimuli (learned or unlearned) occur simultaneously, a motivational conflict emerges that challenges individuals to weigh available options and execute a single behavioral response (avoid or approach). Most previous animal models using approach/avoidance conflicts have often focused on the ability to avoid threats by forgoing or delaying the opportunity to obtain rewards. In contrast, behavioral tasks designed to capitalize on the ability to actively choose to execute approach behaviors despite threats are scarce. Thus, we developed a behavioral test battery composed of three conflict tasks to directly study rats confronting threats to obtain rewards guided by innate and conditioned cues. One conflict task involves crossing a potentially electrified grid to obtain food on the opposite end of a straight alley, the second task is based on the step-down threat avoidance paradigm, and the third one is a modified version of the open field test. We used diazepam to pharmacologically validate conflict behaviors in our tasks. We found that, regardless of whether competing stimuli were conditioned or innate, a low diazepam dose decreased risk assessment and facilitated taking action to obtain rewards in the face of threats during conflict, without affecting choice behavior when there was no conflict involved. Using this pharmacologically validated test battery of ethologically designed innate/learned conflict tasks could help understand the fundamental brain mechanisms underlying the ability to confront threats to achieve goals.

## Introduction

To ensure survival in nature, animals must avoid threats and pursue rewards. This ability involves that animals use inherited or assigned value information of stimuli in the environment (negative or positive valence) to control motivated behaviors (Rangel et al., 2008; Tye, 2018). Traditionally, these two motivational valence systems have been successfully studied separately (Hu, 2016). Defensive and avoidance responses triggered by threats (LeDoux, 2000) have been generally studied separately from approach behaviors elicited by rewards (Cardinal et al., 2002). During foraging, however, animals regularly encounter threats and rewards simultaneously and are consequently challenged to engage in opposing binary choices (avoid or approach) (Choi and Kim, 2010; Hayden and Walton, 2014; Amir et al., 2015; Mobbs et al., 2018). Such a motivational conflict involves a cost–benefit decision determined by the competition processes between these two mutually exclusive systems interacting (Corr, 2013; McNaughton and Corr, 2014). Conflict is elicited when an individual is challenged to make a choice guided by stimuli with opposing valences (threat and reward) to execute a choice between two incompatible behavioral responses (avoid or approach). During such forms of conflict, individuals must either choose to avoid threats at the cost of not benefiting from rewards or to approach rewards at the cost of facing threats. Interestingly, when reaching the choice point, rodents display characteristic oscillatory conflict behaviors that include hesitantly moving back and forward (Miller, 1944), head dips (Takeda et al., 1998), and stretched postures (Grant and Mackintosh, 1963), as if assessing the risks over the decision to make (risk-assessment behaviors).

Conflict behaviors are sensitive to the action of antianxiety drugs (Gray, 1977, 1982; McNaughton and Corr, 2014). Traditionally, approach/avoidance conflict tasks have been useful in validating benzodiazepines like diazepam (DZPM) (Vogel et al., 1971; Rodgers et al., 1997; Calhoon and Tye, 2015). More recent conflict animal models have often focused on studying the decision animals make to avoid threats while forgoing the opportunity or delaying the time to obtain rewards (Moscarello and LeDoux, 2013; Bravo-Rivera et al., 2014; Friedman et al., 2015; Burgos-Robles et al., 2017; Piantadosi et al., 2017; Schumacher et al., 2018; Choi et al., 2019; Miller et al., 2019; Verharen et al., 2019; Walters et al., 2019). Yet, the ability of animals to choose to forage for resources by confronting threats remains understudied and lack a behavioral test battery to comprehensively characterize it.

To directly study animals seeking rewards in the face of threats, we developed three choice-mediated conflict tasks in rodents based on traditional behavioral assays. All of our behavioral tasks involve the comparison of conflict-based vs. no-conflict-based choice behaviors. They differ, however, in that rats must use stimuli with innate and/or acquired negative valences to guide conflict choices. The crossing-mediated conflict task, based on the task used to map self-stimulation brain sites (Olds and Milner, 1954), uses competing cued-conditioned stimuli and involves comparatively assessing the time it takes rats to cross a potentially electrified grid (“threat” zone) to obtain food on the opposite side of a straight alley (“safe” zone) during conflict and no-conflict trials (Bravo-Rivera and Sotres-Bayon, 2020). The step down-mediated conflict task, based on the step-down threat avoidance paradigm, uses conditioned aversive and innate appetitive stimuli. Finally, the foraging-mediated innate conflict task, based on the open field test (Walsh and Cummins, 1976), involves placing food in a brightly lit arena center. We tested the validity of our conflict tasks by administering systemic injections of a commonly used benzodiazepine drug, DZPM. We found that, independently of whether competing stimuli were conditioned or innate, a low dose of DZPM decreased risk assessment and facilitated the ability of rats to actively confront threats, incentivized by reward availability during conflict choice behaviors only.

## Materials and Methods

All procedures were approved by the Institutional Animal Care and Use Committee of the Universidad Nacional Autónoma de México, in compliance with the National Ministry of Health guidelines for the care of laboratory animals. A total of 160 adult male Wistar rats (Instituto de Fisiología Celular breeding colony) 2–3 months of age, weighing 250–300 g were housed in individual polyethylene cages, handled daily to diminish stress responses, and maintained on a standard 12 h light/dark schedule. All experiments were performed during the light phase. To maintain a stable motivation to eat or drink during behavioral training and tests, rats were food restricted (12 g/day of standard laboratory rat chow with a 5-g bonus feeding at the end of each week to maintain rats at 85% of their initial weight) or water restricted (12 ml/day; 6 ml in the morning and 6 ml in the afternoon), respectively.

### Task 1: Crossing-Mediated Conflict Task

**FIGURE 1:**
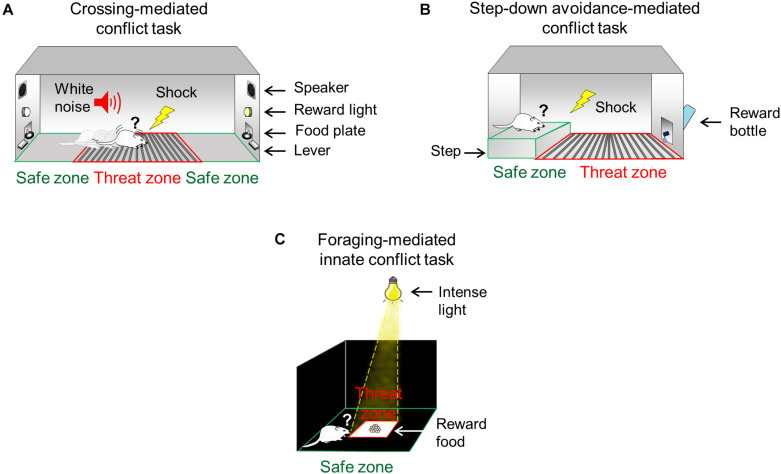
Conflict test battery to study the ability to confront threats in pursuit of rewards. **(A)** During the crossing-mediated conflict task, hungry rats are trained to repeatedly choose to cross a grid (threat zone, red) to press a lever to obtain food (pellets, reward) in the opposite side of the straight alley (safe zone, green), guided by conditioned cues (white noise and light). **(B)** During the step-down avoidance-mediated conflict task, thirsty rats are trained to choose to step down from an elevated platform (safe zone, green) onto a conditioned grid (threat zone, red) to approach a bottle containing sweetened water (saccharin, reward). **(C)** During the foraging-mediated innate conflict task, hungry rats must choose to move from the periphery of the arena (safe zone, green) to enter the intensely illuminated center of the arena (threat zone, red) to obtain food (pellets, reward).

Rats were trained and tested in straight alleys that consisted of acrylic walls with stainless-steel frames (100 cm long × 30 cm wide × 50 cm tall), located in a sound-attenuating cubicle (150 cm long × 70 cm wide × 140 cm tall). The alleys consisted of three zones: two “safe” zones and one “threat” zone (Figure 1A). Each “safe” zone (20 cm long × 30 cm wide) was located on the opposite ends of the alley, and the “threat” zone (60 cm long × 30 cm wide) was located between the two “safe” zones. The floor of the “threat” zone was made of stainless-steel bars (4.8 mm diameter), which delivered a scramble footshock (Coulbourn Instruments, United States), while the floor of “safe” zones was made of acrylic. The “safe” zone included a speaker and standard operant chamber components (cue light, a lever connected to a pellet dispenser, and a food receptacle). The alleys were interfaced with a computer running custom scripts that controlled apparatus hardware (food pellet delivery, cue lights, speakers, and shocker) and recorded task events (lever presses at each end of the alley). The shock grids, the alley floors, and the walls were cleaned with soapy water and 70% alcohol and dried with paper towels between experiments. Prior to conflict training, all rats were trained to press a lever to obtain sucrose pellets (45 mg, dustless precision pellets, Bioserve, United States) in a fixed reinforcement schedule (each lever press was reinforced with one pellet). All sessions started and ended with context-alone exposure (5 min without cue lights, shocks, or noise).

#### Conflict Training

Conflict training involved five succeeding stages: two stages of reward training (reward association and reward crossing), two stages of threat training (threat association and threat crossing), and a final stage of discrimination training to distinguish between conflict and no-conflict trials. Conflict training occurred over a total of 30 days.

##### Reward training

Initially, rats confined to one end of the alley (“safe” zone) using an opaque acrylic wall were trained to associate food availability with a light cue (*Reward association*). A pellet was dispensed to the food receptacle with each lever press when the cue light was illuminated (light trial), while no pellet was delivered in the absence of light (no-light trial). Each light trial ended after a randomly assigned number of rewarded pressing events were achieved (ranging from 5 to 20 presses; a custom script running in a computer associated with the apparatus generated a random number and assigned the type of trial to present), whereas each no-light trial ended after a randomly assigned time had elapsed (ranging from 30 to 180 s). Each reward conditioning session was limited to 30 min, and it involved ∼10 light trials and ∼10 no-light trials, which resulted in ∼15 min per trial type. One session was given per day across 6 days (days 1–6 of conflict training). Next, rats were trained to cross from one end of the alley to the opposite side (from one “safe” zone to the other) to obtain food signaled by light (*Reward crossings*). Short acrylic barriers (9 cm tall) were placed between the grid and “safe” zones to delimit and commit choice-mediated crossing behavior in time and space (choose point). A reward crossing trial started when a light was illuminated in the side of the alley opposite to the location of the rat. The trial ended when the light was turned off, after either the rat had crossed to the opposite “safe” zone and pressed the lever or 180 s had passed without crossing. To promote goal-oriented crosses guided by tracking location of light cue, rather than nonsignaled habitual reactions, rats received one to three reinforced lever presses on the same “safe” zone. Each reward crossing session was composed of 30 trials. One session was given per day for 5 days (days 7–11 of conflict training). Reward training occurred over a total of 11 days (6 days of reward association and 5 days of reward crossing training). By this point, rats had learned that reward crossings involved actively tracking food availability signaled by the cue light in each of the “safe” zones of the alley in the absence of conflict (crossing to obtain food without threat).

##### Threat training

Rats confined to the “threat” zone (middle of the alley) using opaque acrylic walls and a ceiling (similar dimensions to standard classical threat conditioning chambers: 30 cm long, 25 cm wide, 30 cm tall) were trained to associate a white noise (85 dB for 30 s) with a mild footshock (0.5 mA for 1 s) (*Threat association*). To allow the sound coming from opposite sides of the alley to enter the confined conditioning area, the acrylic ceiling was perforated (1.2 cm diameter). Foot shocks were randomly delivered during the 30-s noise presentation to avoid a temporal noise/shock associations that may limit threat-related responses to a specific time (a custom script running in a computer associated with the apparatus generated a random number and delivered the shock). Five noise-shock pairings were delivered per day with a variable intertrial interval of 1–3 min. One session was given per day for 4 days. On the next day, two noise-alone trials (in the absence of shocks) were delivered to test for cued threat memory (days 12–16 of conflict training). Next, rats were trained to cross the alley to obtain food (guided by the light cue), while the threat cue (noise) was presented simultaneously (*Threat crossings*). A threat crossing trial started and ended the same way as a reward crossing trials. The difference between reward and threat crossings was that during threat crossings, rats were challenged to cross to the other side of the alley to obtain food (signaled by the conditioned light) by facing the co-occurring threat (0.5 mA shock signaled by the conditioned noise). Short acrylic barriers (introduced since Reward crossing training) prevented rats from getting exposed to the electrified grid before committing to cross (hesitation at the choice point). The duration of threat crossing trials was increased across days to gradually intensify threat in crossing sessions. Intertrial intervals ranged from 1 to 3 min, and each session was limited to 30 min. One session was given per day for 5 days (days 17–21 of conflict training). Threat crossing trials lasted 30 s in the first 2 days, 60 s on the third day, 90 s on the fourth day, and 120 s on the fifth day of training. Each trial ended either after the time of crossing elapsed (30–120 s) or when rats successfully crossed and pressed the lever on the opposite side of the alley. Threat training occurred over a total of 10 days (5 days of threat association and 5 days of threat crossing training). By this point, rats had learned that threat crossings guided by the simultaneous occurrence of light and sound cues involved conflict (crossing to obtain food despite threat).

##### Discrimination training

The final stage of conflict training consisted of learning to discriminate between no-conflict (crossing to obtain food without threat) and conflict (crossing to obtain food despite threat) trials across 9 days (*Discrimination*). Each discrimination session consisted on a total of 30 crossing trials. Each session was divided into three blocks of 10 trials. Each block of 10 trials consisted of seven or nine no-conflict trials and one or three conflict trials (10 or 30% chance of threat trials, respectively). To prevent rats from predicting types of trials, they were presented in a different sequence across 10-trial blocks (a custom script running in a computer associated with the apparatus generated a random number and assigned the type of trial to present considering the constricted number of types of trials). On each trial, rats were allowed a limited time to choose whether to cross or not to cross the alley (180 s). One session was given per day for 9 days (days 22–30 of conflict training). To gradually and progressively increase the risk of threat crossing, the proportion of conflict trials and intensity of shock increased across days. Conflict crossings were presented with a 10% chance of threat (3 conflicts vs. 27 no-conflict trials) and 0.5 mA shock intensity the first 3 days (22–24 days), the next 3 days (25–27 days) with a 30% chance of threat (9 conflict vs. 21 no-conflict trials) using the same shock intensity as the sessions before, and finally, the last 3 days (28–30 days) with the same percentage of threat occurrence as the session before but with increased intensity of shock of 0.1 mA/day until it reached 0.8 mA at the end of discrimination training. At this point, rats had learned to discriminate between conflict against no-conflict trials. Rats that did not learn to discriminate between types of trials by the end of discrimination training (*p* > 0.05 in the comparison between conflict and no-conflict trials in the last block of trials: 2 out of 18), and rats that did not learn to cross in 180 s were excluded from the study (2 out of 18). Therefore, all trials for all animals included in the study were rewarded.

#### Conflict Test

The conflict test was performed 1 day after conflict training ended. Rats were tested with 10 crossing trials. These trials were presented in the same conditions as the last day of discrimination training (30% chance of threat: three conflict and seven no-conflict pseudorandomly presented trials) but in the absence of shocks. Results were expressed comparing the average of conflict against no-conflict trials before (pre-test) and after (test) injections.

### Task 2: Step-Down Avoidance-Mediated Conflict Task

Rats were trained and tested in a modified step-down inhibitory avoidance chamber (50 cm long × 25 cm wide × 25 cm tall) located in a sound-attenuating cubicle (61.5 cm long × 62.5 cm wide × 65 cm tall). The step-down chamber consisted of two zones: one “safe” and one “threat” zone (Figure 1B). The floor of the “threat” zone (30 cm long × 25 cm wide) consisted of stainless-steel bars (4.8 mm diameter) delivering a scrambled footshock (Coulbourn Instruments, United States), while the floor of the “safe” zone (20 cm long × 23.5 cm wide × 6 cm tall) was an acrylic-covered elevated step platform. An acrylic sliding door was located between the threat and safe zones to allow precise timing of step-down choice behavior (choice point). Compared to the standard step-down avoidance (Izquierdo et al., 1997), the modified chamber includes a bottle with 12 ml of saccharin solution (0.1%) placed on the wall of the “threat” zone opposite from the “safe” zone. This modification represents a strong incentive that motivates the animal to step down from the safe platform. Before the training started, rats were habituated to the step-down behavior in the chamber for 1 day (the bottle was present but empty) and water deprived for 12 h. Training and test sessions started with rats confined to the elevated platform for 5 min in the safe “zone” (the sliding door was closed). Shock grids as well as chamber floors and walls were cleaned with soapy water and 70% alcohol and dried with paper towels between experiments.

#### Conflict Training

Two variants of the step-down task were used in separate groups of rats. One task involved conflict training, while the other task did not involve conflict. The only difference between these tasks is that conflict training involved water-restricted rats (thirsty), while rats in the no-conflict task had free access to water (not thirsty). Both tasks involved reward presentations and threat association training. During reward presentation sessions, after pulling the sliding door, rats were allowed to drink from the bottle placed in the opposite side of the elevated platform (“threat” zone) containing saccharin solution (12 ml). Each reward presentation session was limited to 10 min. One session was given per day for 5 days (days 1–5) to allow familiarization with the chamber, overcoming neophobia to the novel taste (saccharin) and achieving stable reward sampling across days. The following 2 days (days 6 and 7), rats were trained to associate the act of stepping down from the platform with a mild footshock (0.5 mA). This footshock lasted until rats came back to the safety platform. One trial per session was given per day. Step-down training for each of the two tasks (conflict and no-conflict) occurred over a total of 7 days. By this point, two separate groups of rats had been trained in two different conditions: one group (motivated to drink) had learned to step down to obtain sweetened water despite threat (conflict group), while another group (not motivated to drink) had learned to avoid threat by not stepping down (no-conflict group).

#### Conflict Test

A test that involved conflict and another that did not involve conflict were performed 1 day after training ended. Rats were tested in the same conditions of the last day of training of each of the groups (conflict and no-conflict) but in the absence of shocks. Results were expressed comparing the average of conflict and no-conflict group step-down latencies before (pre-test) and after (test) injections.

### Task 3: Foraging-Mediated Innate Conflict Task

Rats were tested in a modified open field arena (90 cm long, 90 cm wide, and 60 cm tall) with walls made of wood and floors made of textured transparent acrylic, located in a dark room. The arena was divided into two zones: one “safe” zone (60 cm long and 60 cm wide) and one “threat” zone (30 cm long and 30 cm wide) (Figure 1C). Compared to the standard open field (Stefanski et al., 1992), our modified arena includes an intense beam of light (1500 lx) focused at the center of the arena (“threat” zone), allowing the periphery of the arena to remain dark (“safe” zone). This modification represents an added innately aversive stimulus to the already innately aversive center of the open field without affecting the periphery of the arena. The floor and wooden walls were cleaned with soapy water and 70% alcohol and dried with paper towels between experiments.

#### Conflict Test

Two types of open field tests were used in separate groups of rats. One test involved conflict, while the other test did not involve conflict. The only difference between these two tests is that the conflict test involved placing 30 sucrose pellets (1.350 g) in the center of the field (“threat” zone) to motivate rats to forage for food (Britton and Britton, 1981), whereas the no-conflict test did not involve food in the arena. Motivation to forage for food was maintained in the periphery by placing five sucrose pellets (0.9 g) on each of the corners of the arena. To avoid innate aversion to the novel taste (neophobia) of pellets, rats received 20 sucrose pellets (1.125 g) in their home cages for 2 days before tests. Rats were individually placed into the “safe” zone, and their time spent in the “threat” and the “safe” zones was recorded for 10 min. The time spent at the center of the open field arena was used to evaluate foraging despite threat (conflict) an the time spent in the periphery was used to evaluated foraging without the threat (no conflict). Test results were expressed comparing the average times spent on either center or periphery of the arena after injections in conflict and no-conflict groups.

##### Open field test

Locomotor activity and anxiety were evaluated in a standard open field arena (no food or intense light involved) (Schmitt and Hiemke, 1998). Rats were individually placed at the center of the open field arena, and their behavior was recorded for 5 min. Distance traveled in the open field was used to evaluate locomotion, and entries at the center of the arena were used to evaluate anxiety-like behavior.

##### Beam walking test

Motor coordination was evaluated using beam walking behavior (Goldstein and Davis, 1990). The beam was a wooden bar (100 cm long × 2 cm wide) placed 80 cm above the floor with an inclination of 15°. An opened home cage was placed at the end of the beam to motivate rats to walk. The latencies to arrive at the end of the beam and enter into the home cage were recorded. Rats were trained for 5 days to walk on the beam toward the home cage, initially (days 1 and 2) starting at a distance of 50 cm and then (days 3–5) starting at a distance of 100 cm from the home cage. Rats were allowed to stay in the home cage for 30 s. The next day, rats were tested on beam walking.

##### Food intake test

Feeding behavior was evaluated by calculating food intake in rats in their home cages (Britton and Britton, 1981; Rex et al., 1996). Food consumption was assessed across 5 days. Each day, rats were presented with 30 g of sucrose pellets placed in a familiar food plate. To calculate food intake, the food plate was weighed before and after 30 min of food presentation.

##### Saccharin intake test

Drinking behavior was evaluated by calculating free intake of saccharin solution (sweetened water) in rats in their home cages. Sweetened water consumption was assessed across 5 days. Each day, rats were presented with a familiar water bottle containing 50 ml of saccharin solution (0.1%). To calculate saccharin intake, the water bottle was weighed before and after 30 min water bottle presentation.

### Systemic Drug Administration

Diazepam (1 or 2 mg/kg of weight) dissolved in a sterile solution (DZPM, 5 mg/ml, Valium^®^, Roche, México) or an equivalent volume of saline solution (SAL) was subcutaneously administered 30 min before all behavioral tests.

### Data Collection and Analysis

All behaviors were recorded with digital video cameras (Provision, model D-380D5) located above each task apparatus. Lever-pressing events were recorded by a computer running MATLAB^®^ (MathWorks Inc.) custom scripts interfaced with the straight alley. Lever-pressing events (presses per minute) during light or no-light trials were expressed as blocks of 10 min. Each session (one per day) consisted of three of these 10-min blocks. Crossing latencies consisted of the total time (seconds) that rats spent to cross to the opposite side of the straight alley and press the lever to obtain food. Latencies to cross were expressed as blocks of 10 trials. Each training session (one per day) consisted of three of these 10-trial blocks, and the test session consisted of one block of 10 trials. Freezing responses were expressed as the percent of time spent without movement (except for respiration) during context alone exposure (5 min baseline before noise presentations) or 60 s after noise presentations. Step-down latencies were expressed as the total time (seconds) rats took, after pulling the sliding door, to step down from the elevated platform with four paws onto the grid. Beam walking latencies consisted of the total time (seconds) spent before arriving at the end of the beam to enter the home cage. Food and sweetened water intake were calculated by comparing food-receptacle or water-bottle weight (grams) before and after 30 min of food bottle presentation. Time spent freezing, as well as step-down, beam walking latencies, food and sweetened water intake, as well as risk assessment responses were manually recorded by trained observers blinded to the experimental conditions. Behavior in the open field arenas was recorded using a tracking software (ANY-maze; Stoelting, United States) and expressed as traveled distance (cm) or time spent (seconds) at the center of the field during 5 or 10 min. The number of risk assessment behavioral events (approach/avoidance oscillations toward the reward site) were counted at the choice point, where, depending on the nature of the task, rats must choose to cross, step down, or forage at the center of the open field. Three different types of risk assessment behaviors were evaluated at the choice point: (1) hesitation to cross the alley, (2) stretched posture when stepping down, and (3) head dipping to the center of the open field arena. During the conflict crossing task, a hesitation event was counted every time a rat touched, with at least two paws, the acrylic barrier dividing the safe and threat zones (choice point) and returned with its four paws to the safe zone without crossing. During the step-down conflict task, a stretched posture event was counted every time a rat reached the border separating the safe and threat zones (choice point) and elongated its body forward by placing only two paws on the grid and returned back with its four paws into the safe platform. Finally, during the foraging conflict task, head-dipping events were counted every time a rat introduced its head into the border of the threat zone (choice point) and completely returned back to the safe zone (open-field periphery). Data from all experiments were first tested for normality using the one-sample Kolmogorov–Smirnov test. After testing for the normality of data, experimental groups were compared by using, when appropriate, Student’s two-tailed *t* tests (paired or unpaired) or analysis of variance (one- or two-way between groups or within-subjects ANOVA) followed by planned comparisons or Tukey’s multiple comparisons *post hoc* tests (STATISTICA; StatSoft, United States).

## Results

To determine which DZPM dose was appropriate to test the validity of our conflict tasks, we started by evaluating two DZPM doses in two commonly used behavioral assays. We injected rats with DZPM at a low dose (1 mg/kg), a high dose (2 mg/kg), or SAL before open field (Supplementary Figure 1A) and beam walking (Supplementary Figure 1B) tests. Using one-way ANOVA (followed by *post hoc* tests, below), we found differences between groups in locomotion, motor coordination but not on anxiety-like behavior, as indicated, respectively, by differences on distance traveled [*F*_(__2,32__)_: 6.49, *p* = 0.004] and beam walking time [*F*_(__2,17__)_: 10.38, *p* = 0.001] but no differences on entries to center [*F*_(__2,31__)_: 1.05, *p* = 0.359]. We found that DZPM at a low dose did not affect locomotion, anxiety-like behavior, nor motor coordination, as indicated, respectively, by similar levels of total distance traveled (SAL, 222.9 cm; DZPM, 217.4 cm; *p* = 0.98) and the number of entries to the center of the open field (SAL, 1.94; DZPM, 1.0; *p* = 0.444), as well as similar times to arrive to the end of a narrow elevated beam, as compared to the control group (SAL, 9.6 s; DZPM, 12.8 s; *p* = 0.99). In contrast, rats injected with a high DZPM dose impaired locomotion and motor coordination, without affecting anxiety-like behavior, as indicated by decreased levels of total distance traveled (SAL, 222.9 cm; DZPM, 103.6 cm; *p* = 0.004) and a similar number of entries to the center of the open field (SAL, 1.94; DZPM, 1.1; *p* = 0.501), as well as increased times to arrive at the end of a narrow elevated beam (SAL, 9.6 s; DZPM, 120.7 s; *p* = 0.003), as compared to the control group. Given the impairment effects on locomotion and motor coordination observed after injecting a high dose of DZPM but not after injecting a low dose (low vs. high DZPM doses: distance traveled in open field, *p* = 0.023; beam walking time, *p* = 0.004), we used a low dose of DZPM to test the validity of conflict behaviors in our tasks. Next, we evaluated the effect of this DZPM dose on two types of choice-mediated conflict behaviors in our three conflict tasks: (1) the time it takes rats to successfully confront threats to obtain rewards and (2) the number of times rats displayed risk assessment responses toward the reward site.

### Diazepam Facilitated Crossings During Conflict Test Without Affecting No-Conflict Behaviors

The crossing-mediated conflict task is a modified version of a task previously used to map self-stimulation brain sites. In a series of hallmark studies, Olds and Milner (1954) used such a task where the reward after crossing the grid was electrical stimulation. Our task involves natural food reward rather than an electrical reward and the use of discrete learned cues to guide choice behavior. Thus, our task requires that a hungry rat, previously trained to press a lever for food cued by light, crosses an electrified grid to get to reach a lever on the other side of the straight alley that triggers a food dispenser. Our task allows for isolation of key variables in the same individual: appetitive drive indicated by latency to approach in safe no-conflict trials, defensive reactive response indicated by freezing in the “threat” zone, and competition between aversive and appetitive drives indicated by latency to approach in conflict trials.

**FIGURE 2:**
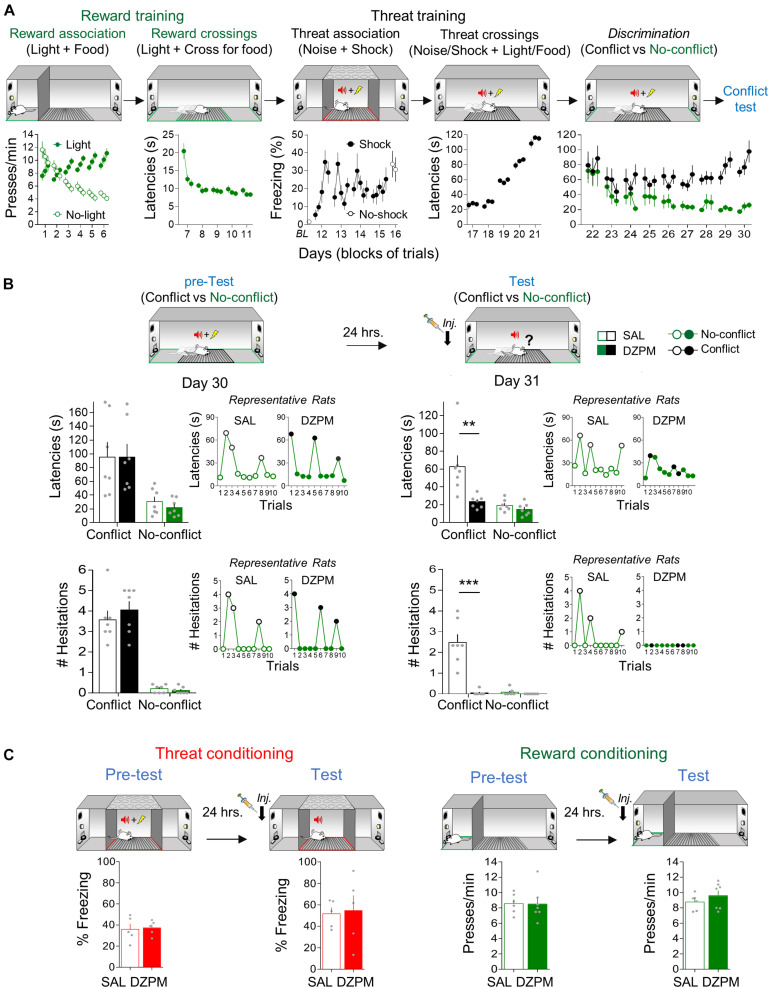
Diazepam decreases crossing latencies and hesitation events during conflict without affecting no-conflict trials. **(A)** Rats acquired crossing-mediated conflict in 30 days (*n* = 14). First, hungry rats, confined to the safe zone (green), learned to associate pressing a lever with food availability cued by a light (reward conditioning), followed by training to cross to the opposite safe zone of the straight alley to obtain food cued by light (no-conflict crossings). Then, rats, confined to the threat zone (grid, red) of the alley, learned to associate the occurrence of white noise with a mild footshock (threat conditioning), followed by training to cross with both learned contingencies (light/food and noise/shock) presented simultaneously (conflict crossings). Finally, rats were trained to discriminate crossing trials guided by no-conflict (light/food alone) or conflict (light/food and noise/shock) cues. Data from lever pressing (per minute) and time to cross to the opposite safe zone of the alley (latency in seconds) are presented in blocks of three trials per day, whereas percent time spent freezing (with or without shock) is presented for each trial. By the end of crossing-mediated conflict training, rats showed high crossing latencies during conflict trials (black) compared to no-conflict trials (green). **(B)** Before injection (pre-test), saline solution and diazepam groups (SAL, *n* = 7; DZPM, *n* = 7) showed similarly high crossing latencies (top) and hesitation events toward the reward site (bottom) during Conflict trials and similarly low crossing latencies and hesitations events during No-conflict (*left*, trials averages of experimental groups; *right*, trial by trial performance of representative rats). The following day, after injection (test), the diazepam-treated rats decreased crossing latencies (top) and hesitation events (bottom) during conflict trials (no shock) while leaving no-conflict trials intact, as compared to the saline-treated rats (*left*, trial averages of experimental groups; *right*, trial by trial performance of the same representative rats shown in pretest). **(C)** Rats were separately trained in threat and reward conditioning tasks. Before (pre-test) and after (test) injection, SAL and DZPM groups (SAL, *n* = 5; DZPM, *n* = 5) showed similar reactive freezing responses during aversive conditioning and numbers of lever presses per minute during appetitive conditioning (SAL, *n* = 6; DZPM, *n* = 7). Error bars indicate Standard Error of Mean (SEM). BL, baseline. **p < 0.01; ***p < 0.001.

To evaluate the effect of DZPM on crossing-mediated conflict behavior at test, rats received conflict training in three consecutive stages: reward and threat trainings, followed by trial discrimination training (Figure 2A). Threat and reward training involved associative and crossing sessions. During reward association sessions (6 days), rats confined to one of the ends of the straight alley (“safe” zone) gradually learned that a light cue was associated with the availability of food after pressing a lever, whereas the absence of light was associated with lack of food availability, as indicated by the progressively decreasing lever-pressing levels in no-light trials as compared to stable lever pressing in light trials across training days [two-way repeated-measures ANOVA, group: *F*_(1,26__)_ = 12.3, *p* = 0.001; trial block: *F*_(17,442__)_ = 6.09, *p* < 0.001; interaction: *F*_(17,442__)_ = 24.0, *p* < 0.001]. Notice that reward conditioning shifted an apparent natural preference for lever pressing during the absence of light cue at the beginning of training [day 1, first trial block: light, 7.58; no-light, 11.63 presses/min; Student’s two-tailed paired *t* test, *t*_(13__)_ = −2.69, *p* = 0.018], to stable pressing preference guided by the light cue at the end of training compared to trials in the absence of light [day 6, session average: light, 10.12; no-light, 4.49 presses/min; Student’s two-tailed paired *t* test, *t*_(13__)_ = 7.79, *p* < 0.001]. During reward crossing sessions (5 days), the alley was opened to allow for rats to learn to track food availability on opposite ends of the alley guided by the light cue, as indicated by a sustained decrease in time spent to choose to cross the alley (latency) across days [one-way repeated-measures ANOVA, *F*_(14,182__)_ = 16.4, *p* < 0.001]. Notice that rats rapidly learned to cross for food cued by light, as indicated by long latencies at the beginning of training (day 7, first trial block: 20.4 s) as compared to short stable latencies by the end of training [day 11, session average: 8.7 s; Student’s two-tailed paired *t* test, *t*_(13__)_ = 6.11, *p* < 0.001].

Threat training was initiated 1 day after reward training. During threat association sessions (5 days), rats, confined to the middle of the alley (“threat” zone), were conditioned and tested. Rats rapidly learned to associate noise presentations with shock occurrence, as indicated by increased freezing levels across the first day of conditioning [day 12, Student’s two-tailed paired *t* test, *t*_(13__)_ = −5.28, *p* < 0.001]. Consistent with this learning, rats showed robust threat memory retrieval elicited by noise in the absence of shock, as indicated by high freezing levels during the test compared to preconditioning baseline levels [day 16, Student’s two-tailed paired *t* test, *t*_(13__)_ = −5.23, *p* < 0.001]. Between initial conditioning and test, rats exhibited escape-like behaviors as indicated by variable freezing levels (days 13–15). Such threat-elicited behavior reflects the unexpected shock delivery timing during noise presentations (see section “Materials and Methods”) intended to prime active, rather than passive, defensive responses when challenged to cross the alley. Starting the next day, the alley was opened to allow crossings. During threat crossing training (5 days), rats learned to cross to the opposite end of the alley to obtain food despite an electrified grid signaled by noise presentations, as indicated by increased crossing latencies across days [one-way repeated-measures ANOVA, *F*_(14,182__)_ = 142.4, *p* < 0.001]. By gradually and progressively increasing the duration of time allowed to cross the grid across days (see section “Materials and Methods”), rats learned to limit threat crossing response timing in the choice point, as indicated by short latencies in the beginning (day 17 first trial block: 26.1 s) compared to long latencies at the end of training [day 21 session average: 113.3 s; Student’s two-tailed paired *t* test, *t*_(13__)_ = −16.6, *p* < 0.001].

These results indicate that, at this point, rats had learned reward and threat contingencies separately. In the final conflict training stage, rats received discrimination training (9 days). Rats learned to distinguish, in the same session, between pseudorandomly presented crossing trials that involved conflict (light and noise presented simultaneously) against those that occurred in the absence of conflict (light without noise), as indicated by increasingly long latencies during conflict and low latencies during no-conflict trials across days [two-way repeated-measures ANOVA, group: *F*_(1,15__)_ = 34.2, *p* < 0.001; trial block: *F*_(26,390__)_ = 2.72, *p* < 0.001; interaction: *F*_(26,390__)_ = 1.53, *p* = 0.047]. Notice that rats started training with similar latencies between trial types [day 22 session trial averages: conflict, 94.0 s vs. no-conflict, 78.8 s; Student’s two-tailed paired *t* test, *t*_(13__)_ = −1.66, *p* = 0.119] and ended (day 30 session average) with prominently high latencies during conflict trials (81.6 s) as compared to low latencies during no-conflict trials [22.7 s; Student’s two-tailed paired *t* test, *t*_(13__)_ = −8.00, *p* < 0.001].

To evaluate the effect of DZPM on crossing-mediated conflict behavior at test, rats were separated into two groups matched by similar crossing latencies within trial types and maintained differences between trial types [test, factorial, ANOVA, pre-test, group: *F*_(1,24__)_ = 0.07, *p* = 0.787; trials: *F*_(1.24__)_ = 20.65, *p* < 0.001; interaction: *F*_(1,24__)_ = 0.076, *p* = 0.784] (Figure 2B, top). The next day (day 31), rats were tested with 10 crossing trials, in the absence of footshock, after SAL or DZPM injections. Notably, we found that DZPM decreased crossing latencies during conflict test without affecting no-conflict trials, as indicated by low latencies in DZPM-injected rats (23.3 s) compared to long latencies in saline-injected rats (62.6 s) during conflict trials, yet similar latencies between groups during no-conflict trials (DZPM, 14.7 s; SAL, 19.4 s) [test, factorial, ANOVA, test, group: *F*_(1,24__)_ = 10.6, *p* = 0.003; trial block: *F*_(1,24__)_ = 14.75; *p* < 0.001; interaction: *F*_(1,24__)_ = 6.58; *p* = 0.016]. Notice that the DZPM-treated rats crossed as rapidly when conflict was involved as when the conflict was absent, as indicated by similar latencies in conflict as compared to no-conflict trials during test after DZPM injection (23.3 and 14.7 s latencies, respectively; *post hoc* comparison after ANOVA: *p* = 0.80).

In addition to the crossing latencies, we evaluated the effect of DZPM on risk assessment behavior evaluated by the expression of hesitation events in the conflict and no-conflict trials (Figure 2B, bottom). Before injection, both saline and DZPM groups showed similarly high numbers of hesitation events in conflict trials and similarly low number of hesitation events during no-conflict trials [pretest, factorial ANOVA, group: *F*_(1,24__)_ = 0.40, *p* = 0.53; trials: *F*_(1.24__)_ = 137.6, *p* < 0.000; interaction: *F*_(1,24__)_ = 0.80, *p* = 0.378]. The next day, after injection, we found that DZPM blocked the expression of hesitation events during the conflict test without affecting no-conflict trials, as indicated by almost a complete lack of hesitation events displayed by DZPM-injected rats (0.047 events) compared to high amount of hesitation events displayed by the saline-injected rats (2.47 events) during conflict trials [test, factorial ANOVA, group: *F*_(1,24__)_ = 38.42, *p* = 0.000; trial block: *F*_(1,24__)_ = 36.36; *p* < 0.000; interaction: *F*_(1,24__)_ = 33.58; *p* = 0.000], yet similar hesitation events between groups during no-conflict trials (DZPM, 0.00 events; SAL, 0.081 events). Thus, consistent with the effect on crossing latencies, although DZPM decreased risk assessment behavior during conflict trials, it did not affect no-conflict trials (0.00 and 0.04 hesitation events, respectively, *post hoc* comparison after ANOVA: *p* = 0.99). Surprisingly, even though DZPM decreased both the crossing latencies and the risk assessment behavior only during conflict trials, these behavioral responses appear to be independent of each other, as indicated by lack of correlation between them in both experimental groups (Pearson correlation test, SAL, *R* = 0.49, *p* = 0.25; DZPM, *R* = 0.28, *p* = 0.53; for example, see rat 3 for lack of direct relationship between latencies and hesitations in Supplementary Figure 2). Figure 2B insets and Supplementary Figure 2 show trial by trial performance comparison of individual representative rats before (pre-test) and after (test) injection of either SAL or DZPM, highlighting that DZPM facilitated alley crossings and abolished risk assessment behavior that involves selecting the appropriate choice behavior (approach a reward despite threat) guided by competing aversive and appetitive memories.

To test whether this DZPM effect could be explained by impairment on the expression of threat and/or reward memories independently, we injected SAL or DZPM in separate groups of rats before threat and reward conditioning retrieval tests (Figure 2C). Separate groups of rats were subjected to threat conditioning and reward conditioning as above (see section “Threat Association and Reward Association Training”, respectively) and tested for memory retrieval on the last day after injection of either DZPM or SAL. During threat and reward training (pre-test), both saline- and DZPM-injected rats showed similarly high levels of freezing and lever pressing [Student’s two-tailed unpaired *t* test, *t*_(8__)_ = −0.21, *p* = 0.83; *t*_(11__)_ = 0.06, *p* = 0.95]. After injection (test), we found that DZPM did not affect threat and reward memory expression, as indicated by similar freezing (DZPM, 57.33; SAL, 51.6% freezing) and lever-pressing levels (DZPM, 8.7; SAL, 9.6 presses/min) compared to their respective saline groups during the retrieval test [Student’s two-tailed unpaired *t* test, *t*_(8__)_ = −0.49, *p* = 0.63; *t*_(11__)_ = −1.03, *p* = 0.32]. Finally, we evaluated whether crossing-mediated choice behavior during the conflict test was goal directed or habitual by retraining rats for 3 days, giving rats free access to food for a day and the following day evaluated for crossing behavior. We confirmed that crossing behavior was guided by goals and not by habits, as indicated by maintained trial discrimination but increased latencies in both conflict and no-conflict trials in satiated (conflict trials, 154 s; no-conflict trials, 59 s) vs. no-satiated rats (conflict trials, 64.6 s; no-conflict trials, 17 s) during test [comparison: conflict trials, Student’s two-tailed paired *t* test, *t*_(13__)_ = −5.43, *p* < 0.001, no-conflict trials, Student’s two-tailed paired *t* test, *t*_(13__)_ = −684, *p* < 0.001]. These results indicate that DZPM does not affect threat- and reward-related behaviors *per se* but rather facilitates goal-directed conflict behaviors that involve simultaneously occurring threat and reward-predicting stimuli that compete for the selection of the appropriate choice behavior. Thus, taken together, these findings validate our crossing-mediated conflict task by showing that DZPM facilitates the ability of rats to choose to confront learned threats to execute learned motivational responses that lead to obtaining food.

### Diazepam Facilitated Step-Down Latencies During Conflict Test Without Affecting No-Conflict Behaviors

Innate stimuli often guide the act of facing threats to obtain rewards. Animals often forage for rewards that possess innate value, such as water and sweet tastes. To study animals seeking an innate reward in the face of a learned threat, we evaluated retrieval of an aversive memory in two variants of the step-down task, conflict (with thirsty rats) and no-conflict (with no-thirsty rats). In the standard step-down avoidance task, rats rapidly learn stepping down from a safe platform that predicts a footshock in an electrified grid floor (Izquierdo et al., 1997). To study conflict behavior, we modified this task by placing a bottle containing naturally preferred sweetened water at the end of the grid, opposite to the safe platform (see section “Materials and Methods”). This task is similar to one recently used to study sucrose-seeking behavior (Nieh et al., 2015) but differs in that: (1) rats are restrained by a sliding door at the choice point to delimit choice-mediated behavior in time and space and (2) the critical test assay occurs in the absence of footshocks to avoid potential pain confounds.

**FIGURE 3:**
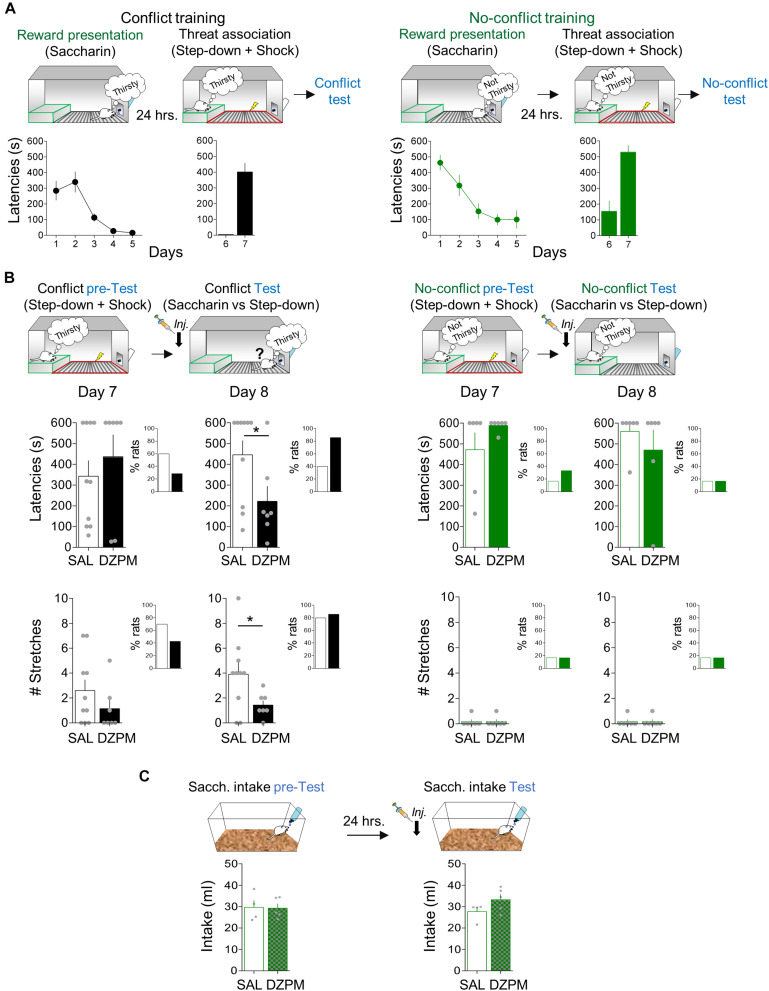
Diazepam decreases step-down latencies and stretched postures during the conflict test without affecting no-conflict conditions. **(A)** Rats acquired step-down avoidance either mediated by conflict or no-conflict. *Left*, In the conflict condition (black), rats (*n* = 17) innately motivated to drink (thirsty) step down from the platform (safe zone, green) to obtain sweetened water (saccharin; reward presentation) from the bottle at the end of the grid, followed by learning that the act of stepping down was associated with the occurrence of a mild footshock in the grid context (threat zone, red). *Right*, In the no-conflict condition (green), rats (*n* = 12) with free access to water (not thirsty) stepped down the platform to obtain saccharin solution, followed by learning that stepping down was associated with footshock delivery in the grid. Time to step down with four paws onto the grid (latency in seconds) is presented by a single trial per day. By the end of the training, both groups showed high latencies to step down. **(B)** Before injection (pre-test), SAL and DZPM groups, in both conflict (*left*, black) and no-conflict (*right*, green) conditions, showed high latencies to step down (top) and stretched postures (stretches, bottom) toward the reward site. The following day, after injection (test), DZPM-treated rats decreased step-down latencies (top) and the numbers of stretches (bottom) during the conflict condition (*n* = 7) without affecting the no-conflict condition (*n* = 6), as compared to the SAL-treated rats (*n* = 10 and *n* = 6, respectively). The inset graphs show the percent of rats that successfully stepped down from the platform to approach the reward [latencies (top) and the rats that displayed stretches (bottom)] during both conflict and no-conflict conditions before (pre-test) and after (test) drug manipulation. **(C)** Rats in their home cages showed similar levels of saccharin intake before (pretest) and after (test) injection of SAL and DZPM (SAL, *n* = 4; DZPM, *n* = 5). Error bars indicate SEM. *p < 0.05.

To evaluate step-down avoidance-mediated conflict behavior at test, we compared choice behavior (latencies to step down or not to step down the platform to obtain water with saccharin) in two separate groups of rats. One group of rats received conflict training, while the other group received no-conflict training. Conflict training involves rats motivated to face the learned threat because they are thirsty, whereas no-conflict training involves rats with free access to water (satiated) and therefore were not motivated to step down the safe platform. Both groups of rats received conflict and no-conflict training in two consecutive stages: reward presentation and threat training (Figure 3A). During reward presentation (5 days), rats gradually learned that at the end of the grid, there was a bottle of water containing saccharin, as indicated by the progressively decreasing latencies in both conflict [one-way repeated-measures ANOVA, *F*_(1,64__)_ = 17.63; *p* < 0.001] and no-conflict training [one-way repeated-measures ANOVA, *F*_(1,44__)_ = 11.35; *p* < 0.001]. In a few days, rats familiarized with the box and identified the location of the reward, as indicated by long latencies in the first reward presentation (day 1, conflict training: 284.4 s; day 1, no-conflict training: 463.0 s) as compared to short latencies by the last reward presentation session [day 5, conflict training: 15.5 s; day 5, no-conflict training: 101.0 s; conflict training: Student’s two-tailed paired *t* test, *t*_(16__)_ = 4.53, *p* < 0.001; no-conflict training: *t*_(11__)_ = 5.04, *p* < 0.001]. At this point, both groups of rats showed similar step-down latencies to obtain saccharin [Student’s two-tailed unpaired *t* test, *t*_(27__)_ = −1.73, *p* = 0.094].

Threat association was initiated 1 day after the reward presentation. During threat association sessions (2 days), rats received a mild footshock in the grid (“threat” zone) immediately after they stepped down from the platform (“safe” zone). Rats rapidly learned to associate the action of stepping down from the platform with footshock occurrence, as indicated by short latencies in the beginning (day 6, conflict training: 4.1 s; day 6, no-conflict training: 154.0 s) compared to long latencies at the end of training in both group of rats [day 7, conflict training: 401.0 s; Student’s two-tailed paired *t* test, *t*_(16__)_ = −6.97, *p* < 0.001; day 7, no-conflict training: 529.9 s; *t*_(11__)_ = −5.64, *p* < 0.001]. At this point, rats had associated the step-down response with footshock.

To evaluate the effect of DZPM on step-down avoidance-mediated conflict behavior at test, rats that received conflict or no-conflict training were separated into two groups matched by similar step-down latencies [day 7, pre-test, conflict group: Student’s two-tailed unpaired *t* test, *t*_(15__)_ = −0.74, *p* = 0.46; day 7, pretest, no-conflict group: *t*_(10__)_ = −1.40; *p* = 0.19; Figure 3B, top]. The next day (day 8), rats were tested in a single trial without footshock after SAL or DZPM injections. Notably, we found that DZPM decreased step-down latencies during the conflict test, as indicated by low latencies in DZPM-injected rats compared with saline-injected rats [SAL, 446.1 s; DZPM, 225.5 s; Student’s two-tailed unpaired *t* test, *t*_(15__)_ = 2.2; *p* = 0.043]. In contrast, DZPM did not affect step-down latencies during no-conflict test, as indicated by similar long latencies in diazepam-injected rats (s) compared with the saline group [SAL, 560.3 s; DZPM, 470.1 s; Student’s two-tailed unpaired *t* test, *t*_(10__)_ = 0.85; *p* = 0.41]. Consistent with the effect of DZPM on step-down latencies, we observed a greater proportion of DZPM-injected rats (85%), as compared to saline-injected rats (40%), that stepped down pursuing reward during the conflict test, while saline- and DZPM-injected rats stepped down similarly (SAL 16.6%; DZPM, 16.6%) during the no-conflict test (Figure 3B, top, insets).

In addition to the step-down latencies, we assessed a risk assessment behavior, evaluated by the expression of stretched posture events in the conflict and no-conflict groups (Figure 3B, bottom). Before injection (pre-test), both saline and DZPM groups showed similarly high number of stretched posture events during the conflict condition and similarly low number of stretched posture events during no-conflict condition [day7, pre-test, conflict group: Student’s two-tailed unpaired *t* test, *t*_(15__)_ = 1.21, *p* = 0.24, no-conflict group: *t*_(8__)_ = 0.00, *p* = 1.0]. Consistent with the step-down latencies, we observed that DZPM decreased the expression of risk assessment behavior during conflict test, as indicated by the lower number of stretched posture events in the DZPM-treated rats as compared with the saline-treated rats [SAL, 3.90 events; DZPM, 1.42 events; Student’s two-tailed unpaired *t* test, *t*_(15__)_ = 2.13, *p* = 0.049] but without increasing the proportion of rats that successfully stepped down pursuing reward (SAL, 80%; DZPM, 85%) (Figure 3B, bottom, inset). In contrast, DZPM did not affect the expression of risk assessment behavior in the no-conflict test, as indicated by similarly low numbers of stretched posture events in both saline and DZPM groups [SAL, 0.16 events; DZPM, 0.16; Student’s two-tailed unpaired *t* test, *t*_(10__)_ = 0.00, *p* = 1.0]. Thus, DZPM injection decreased both crossing latencies and a risk assessment behavior only during the conflict test. Finally, consistent with results from our crossing task, step-down latencies and risk assessment events were not correlated (Pearson correlation test, DZPM, *R* = −0.042, *p* = 0.33; SAL, *R* = 0.058, *p* = 0.87), suggesting two independent choice-related behaviors triggered by conflict.

To further test whether the low DZPM dose effect on conflict behaviors (latencies and hesitation) could be explained by facilitating spontaneous drink seeking, we injected saline or DZPM in a separate group of rats that never received a shock, before a free a saccharin intake test in their home cage (Figure 3C). This nonshocked group is important to test whether DZPM affects reward intake by itself (such as tested in the previous task with a lever-pressing test). Before injections (pre-test), both groups showed similar baseline levels of sweetened water intake in their home cages [SAL, 29.67 ml; DZPM, 28.24 ml; Student’s two-tailed unpaired *t* test, *t*_(7__)_ = 0.34 *p* = 0.74]. After injection during (test), we found that DZPM did not increase saccharin intake, as indicated by similar levels of sweetened water consumed compared to the saline-treated group [SAL, 27.7 ml; DZPM, 33.3 ml; Student’s two-tailed unpaired *t* test, *t*_(7__)_ = −1.67, *p* = 0.13]. These results indicate that DZPM decreases step-down avoidance responses (latencies and risk assessment behavior) during conflict without affecting reward-seeking behavior per se). Thus, taken together, these findings validate our step-down avoidance-mediated conflict task by showing that DZPM facilitates the ability of rats to choose to overcome a learned defensive response to actively obtain a naturally preferred reward.

### Diazepam Facilitated Innate Foraging Behavior During Conflict Test Without Affecting No-Conflict Behaviors

In nature, foraging behavior often requires that animals overcome the risk of encountering a predator. For example, rodents may be challenged to forage for food in an open field despite the risk of being detected by a flying predator. To study the ability of rodents to face innate threats to obtain food, we evaluated foraging behavior in conflict vs. no-conflict-based open field tests. In the standard open field test, rats explore the periphery of a novel environment (“safe” zone) while innately avoiding the center of the open field arena (“threat” zone) (Prut and Belzung, 2003). To study an overt conflict behavior, we developed a modified version of the open field test by placing food in a brightly lit center arena (see section “Materials and Methods”).

**FIGURE 4:**
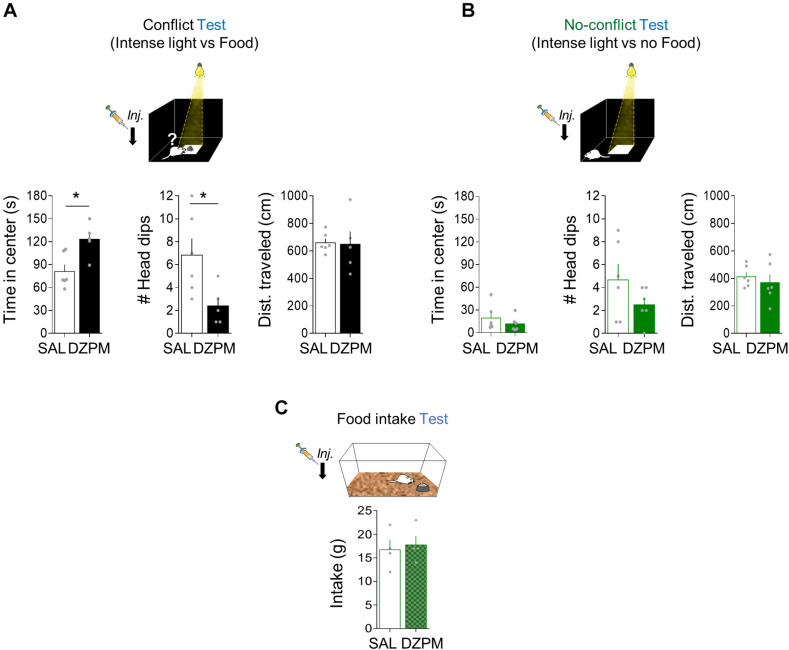
Diazepam increases risky foraging while decreasing head dips during conflict without affecting no-conflict behaviors. Rats placed in an open field arena were tested for innate exploratory behaviors (foraging), head dips (risk assessment), and general locomotion (distance traveled) mediated either by conflict or no-conflict conditions. **(A)** In the foraging-mediated innate conflict test, the diazepam-treated rats spent more time foraging for food (reward) in the brightly illuminated center of the arena (threat zone) while showing low number of head dips events and similar distance traveled in the open field, as compared to the saline group (SAL, *n* = 6; DZMP, *n* = 5). **(B)** In the foraging test where there is no food available (no reward) at the center of the arena (no conflict), time in the brightly illuminated center, head-dip events, and distance traveled were similar in SAL and DZPM groups (SAL, *n* = 6; DZPM, *n* = 6). **(C)** Rats in their home cages showed similar food intake levels after SAL and diazepam injection (SAL, *n* = 4; DZPM, *n* = 4) during the food intake test. Error bars indicate SEM. *p < 0.05.

To evaluate innate foraging conflict behavior, we compared open-field behavior (time spent at the center of an open field arena) in two separate groups of rats. One group of rats was exposed to conflict conditions (Conflict), while the other group was exposed to the same environment in the absence of conflict (No-conflict). Conflict test involves food at the center of the open field arena (Figure 4A), whereas the no-conflict test does not (Figure 4B). To evaluate the effect of DZPM on instinctive foraging conflict behavior, we compared open-field behavior in conflict vs. no-conflict-mediated open field tests after SAL and DZPM injections. We found that DZPM increased foraging behavior for food during conflict test as indicated by more time spent at the center of the open field (“threat” zone) in DZPM-injected rats as compared to saline-injected rats [SAL, 81.03 s; DZPM, 123.16 s; Student’s two-tailed unpaired *t* test, *t*_(__9__)_ = −3.1, *p* < 0.011]. Similar to the other two tasks described before, we found that DZPM decreased risk assessment behavior during the conflict test, as indicated by less head-dipping events in the DZPM-injected rats as compared to the saline-injected rats [SAL, 6.83 events; DZPM, 2.4 events; Student’s two-tailed unpaired *t* test, *t*_(9__)_ = 2.55, *p* < 0.031]. DZPM effect on conflict test was independent of locomotion behavior, as indicated by similar traveled distance (cm) in the saline- and DZPM-injected rats during the conflict test [SAL, 658.73 cm; DZPM, 648.05 cm; Student’s two-tailed unpaired *t* test, *t*_(9__)_ = 0.11, *p* < 0.908]. Consistent with our previous tasks (crossing and step-down mediated conflict tasks), time spent at the center (treat zone) and head-dipping events (risk assessment) were not correlated (Pearson correlation test, DZPM, *R* = −0.70, *p* = 0.11; SAL, *R* = 0.54, *p* = 0.34), suggesting that these are independent choice behavior variables elicited by conflict. In contrast, DZPM did not affect foraging behavior or general locomotion during the no-conflict test, as indicated by both DZPM - and saline-injected rats spending similar time at the center [SAL, 19.3 s; DZPM, 11.7 s; *t*_(10__)_ = 0.96, *p* = 0.35], similarly low head-dipping events [SAL, 4.66 events; DZPM, 2.50 events; *t*_(10__)_ = −1.43, *p* = 0.18] and not differences in the traveled distance [SAL, 412.87 cm; DZPM, 369.35 cm; Student’s two-tailed unpaired *t* test, *t*_(10__)_ = 0.64, *p* = 0.53] in the open-field arena. This DZPM effect on conflict was not due to increased food intake, as indicated by similar food consumed levels compared to the saline group during the test [SAL, 2.25 g; DZPM, 2.20 g; Student’s two-tailed unpaired *t* test, *t*_(__9__)_ = 1.10, *p* = 0.531].

To further test whether the low DZPM dose effect could be explained by hyperphagia (Johnson, 1978; Naruse and Ishii, 1995), we injected saline or DZPM in separate groups of rats before the food intake test in their home cage (Figure 4C). We found that DZPM did not increase food intake, as indicated by similar levels of food consumed compared to the saline-treated group on the third day of the food intake test [SAL, 16.7 g; DZPM, 17.7 g; Student’s two-tailed unpaired *t* test, *t*_(6__)_ = −0.35, *p* = 0.73]. Consistently, by comparing food intake in the same individual during a drug-free test (day 4) and after the DZPM test (day 5), we found that DZPM did not affect food intake, as indicated by the similar weight of food-containing plates between days [day 4 (without drug), 15.0 g; day 5 (with drug), 17.7 g; Student’s two-tailed unpaired *t* test, *t*_(3__)_ = −1.06, *p* = 0.36]. These results indicate that DZPM increases foraging behavior during conflict without affecting no-conflict behaviors (including feeding). Taken together, these findings validate conflict behavior in our open field-mediated conflict task by showing that DZPM facilitates the ability of rats to choose to confront innate threats to forage for food.

## Discussion

We developed three conflict tasks for studying how rodents choose to confront threats to obtain rewards emulating real-life situations. We found that, regardless of whether competing cues were conditioned or innate, DZPM facilitated taking action to face threats by biasing choice toward reward-seeking behaviors during conflict. By using systemic pharmacological manipulations, we show that each of our choice-based tasks are valuable behavioral tools to study conflict behavior. Taken together, our three tasks may be a useful test battery to investigate the underlying brain mechanisms and key neural circuits involved in conflict behaviors associated with facing threats in pursuit of rewards.

### Conflict Tasks

Most previous conflict research has focused on cost–benefit decisions that involve choice between reward options (risky large rewards or small safe rewards), delaying or punishing rewards, thereby biasing behavior against risky reward-seeking behavior and toward avoidance responses (Goette et al., 2015; Jean-Richard-Dit-Bressel and McNally, 2015; Orsini et al., 2016; Piantadosi et al., 2017). Conditioned suppression paradigms have been traditionally used to decrease the occurrence of instrumental behavior (e.g., lever pressing) while the unconditioned aversive stimuli (e.g., footshock) is present (Estes and Skinner, 1941). Under this procedure, animals learn to optimally execute lever pressing when the footshock is not present. In contrast, although based on the same principle, our three conflict tasks are set up so that animals learn that they must suppress prepotent defensive behaviors (learned or innate) to face a threat and thereby obtain a reward (i.e., reward the act of confronting threats), thereby biasing choice toward approach behaviors despite avoidance reactions. Together, our three tasks conform a detailed behavioral test battery useful to evaluate conflict choice behaviors. Our test battery involves tasks that evaluate rewarding risky behaviors based on training rats to distinguish between safe and risky locations to execute a choice behavior. Rats can choose between staying in a safe location to avoid a threat or move to risky location and obtain a reward. To obtain rewards, rats must confront threats. One of our tasks involves extensive training to reward risky crossings, another task involves little training to reward risky step-down behavior, and a final task involves innate behaviors that reward risky foraging for food. On each task, levels of conflict are manipulated differently [each task uses different internal states of the animal (hungry or thirsty)], and different sets of stimuli (tone, light, or context) are used to guide conflict behaviors (Supplementary Table 1). Each individual task provides limited information regarding effects on conflict, but comparing them with no-conflict conditions and control experiments isolating the different motivational components, the results across tasks provide a comprehensive understanding of how a pharmacological manipulation affects conflict. We showed that all conflict behaviors in our tasks are sensitive to DZPM, without affecting no-conflict responses (reactive freezing responses and reward-seeking behaviors), but other experimental manipulations may help dissociate how the nature of the stimuli that guide conflict behaviors (learned and/or innate) underlie distinct cortical and/or subcortical computations in the brain.

Conflict tasks that contrast conflict against no-conflict conditions are not common, and a conflict test battery that comprehensively evaluates and contrasts conflict behaviors is lacking. An advantage of our conflict test battery is that each of the individual tasks used in this toolset allows contrasting choice behaviors in conditions that involve conflict with those that occur in the absence of conflict. In our conflict test battery, the conflict against no-conflict comparison and use of discrete learned cues allowed us to isolate the effects of experimental manipulations on conflict behavioral responses in the same individuals (conflict vs. no-conflict trials in the same rats) and across groups of rats (conflict and no-conflict tests in separate groups of rats). Two separate, recently developed, conflict tasks have also focused on comparing of conflict vs. no-conflict conditions. One task involves a single-trial test on a radial maze, which evaluated rats that choose to either enter an arm in a maze that is associated with competing appetitive and aversive continuous contextual cues (conflict) or entering an arm that was not associated with any cued valences (neutral) (Nguyen et al., 2015). Unlike this task, our crossing-mediated conflict test involves discriminating between discretely timed cues across several trials that involve conflict from those that do not, which may be useful to record the precise timing of changes in neuronal activity with respect to choice behavior (choice point) where animals must commit to suppress defensive responses (or not) to obtain food. A second conflict task that contrasts conflict with no-conflict conditions focused on the expression of freezing defensive response during reward availability (conflict trials) compared to neutral trials (no-conflict) (Burgos-Robles et al., 2017). Unlike this task, our tasks focus on active (rather than passive) suppression of defensive responses to obtain rewards during the conflict, which simulates real-life challenges more readily than other tasks (facing threats driven by foraging behavior). In addition, contrasted with predator-based conflict models (Choi and Kim, 2010; Kimm and Choi, 2018; Walters et al., 2019), the use of discrete conditioned signals in the crossing-mediated conflict task allows precise timing of choice behaviors triggered by threat and reward cues. Taken together, our behavioral test battery (composed of our three tasks and control experiments) allow for the separation of discrete variables controlling behavior in a drive competition setting in the same individual (separation of crossing behaviors that involve conflict and those that lack conflict) or in separate groups of animals (step-down avoidance memory and foraging behaviors mediated by conflict or lack of conflict). Thus, our conflict test battery may represent a valuable tool to comprehensively study the key brain mechanisms that allow animals to seek rewards despite threats.

### Diazepam During Conflict Tests

Because conflict is given by the nature of the tasks in which the risky reward approach is pitted against safety seeking, animals engage in both reward-seeking and threat-elicited behaviors, such that there must be a system that balances these two competing drives (Gray, 1982; McNaughton and Corr, 2014). Perhaps not as common as skewed conflict resolution behaviors (reactive freezing, pure approach, or pure avoidance), intermediate levels of competition between opposing drives involve optimal conflict appraisal (Corr, 2013), which are sensitive to anxiolytic drugs (Gray, 1977, 1982). These intermediate conflict levels take more computational time and are reflected by risky approach behaviors at the choice point as evaluated in this study. DZPM injection, in all of our tasks, allowed rats to reach their goal faster (i.e., to obtain rewards) and with less vacillation to deliberate on the decision to make (i.e., to confront threats). Yet, because these two choice behaviors (goal-directed choice response and decision after risk assessment) appear to be independent from each other, further studies are necessary to understand how conflict elicited by threats influences different goal-directed choice behaviors.

Our finding that DZPM facilitates confronting threats to obtain rewards is consistent with the notion that this benzodiazepine reduces threat-related responses in overt conflict with reward-seeking behaviors. Previous conflict works have shown that DZPM increases: time spent in the open arms of elevated plus maze (Rex et al., 1996; Chaouloff et al., 1997; Dalvi and Rodgers, 1999), time spent at the center of an open field that includes food (Britton and Britton, 1981; Bodnoff et al., 1989; Rex et al., 1996), time spent in an illuminated but not a dark compartment (Chaouloff et al., 1997), foraging behavior (Walters et al., 2019), as well as increased rates of punished reward responding (Vogel et al., 1971; Paterson and Hanania, 2010) and conditioned suppression during conflict (Kilts et al., 1981; Commissaris and Rech, 1982). Engaging in a situation that is simultaneously threatening and rewarding leads to increased physiological arousal (Barker et al., 2019), which may represent an aversive signal (Dreisbach and Fischer, 2012) and thereby induce anxiety. Thus, previous results along with our present findings using conflict tests are consistent with the notion that DZPM may reduce the inability to engage in reward-seeking behaviors (reduce behavioral inhibition) possibly by reducing the increase in arousal that is associated with conflict (“anticonflict effect”) (Liljequist and Engel, 1984; Pericic and Pivac, 1996; Rowlett et al., 2006), thereby allowing the individual to reach their goal. Such anticonflict effect of DZPM on behavioral inhibition has been theorized to represent an effect on a core component of anxiety (Gray, 1977, 1982).

Although the anticonflict effect has been interpreted also as anxiolytic effect (anxiety reducing) (Ljungberg et al., 1987; Beck and Fibiger, 1995), we found that a low dose of DZPM distinctly affects choice behavior during conflict while leaving no-conflict behaviors intact (including anxiety-like behaviors, locomotion, motor coordination, lever pressing, feeding and drinking intake, or reactive freezing defensive responses). Our findings are consistent with the notion, based on varying levels of threat imminence (distance to threat), that conflict behaviors are sensitive to anxiolytic drugs while no-conflict behaviors are not (McNaughton and Corr, 2004). Urgent responses to threats elicit reactive behaviors that are not responsive to anxiolytics, whereas not urgent responses to threats elicit risk-assessment behaviors that are responsive to antianxiety drugs. Our DZPM effect exclusively on conflict contingencies suggests that this drug reduces the probability of engaging predominant, but not urgent, threat-related behaviors only when conflict with reward stimuli is involved. Furthermore, this DZPM effect on conflict behaviors may be most effective at reducing anxiety elicited by anticipation of potential threats (anticipatory anxiety) specifically related to the decision that emerges when confronting threats. In our tasks, challenging rats to confront threats to obtain a reward may induce such an anxiety core component that is triggered by conflict behavior at the choice point and reduced by DZPM. Thus, our findings support the idea that DZPM may have an anticonflict effect by reducing the anxiety trigged by conflict and highlight the possibility of dissociating anxiety related to conflict and no-conflict choice situations.

### Behavioral Strategies to Face Threats

Facing threats to obtain a reward may involve suppressing distinct defensive response strategies in pursuit of a goal. Prior work on suppression of defensive responses has focused on extinction of threat, a passive process (Sotres-Bayon and Quirk, 2010). However, the passive suppression that occurs in extinction is slow and temporary. Threat extinction can take hours to days to reach low (preconditioning) defensive response levels. Once defensive responses to threats have extinguished, behavioral responses triggered by threat can readily return (relapse) with the passage of time (spontaneous recovery), change in context (renewal), or a harmful reminder (reinstatement) (Bouton, 2004). This has led to the investigation of alternative approaches to study regulation of different defensive strategies to face threats (Bravo-Rivera and Sotres-Bayon, 2020). One option is to study the active (rather than passive) goal-directed suppression of defensive responses when facing threats. Novel behavioral paradigms in animals are required to understand the neurobiology of using rewards as incentives to face threats. Moreover, conflict work may benefit from a detailed test battery to evaluate conflict behaviors. In the three main tasks included in our conflict test battery, rats must quickly suppress defensive responses triggered by threats (conditioned or innate) to obtain a reward. Thus, our tasks represent a useful detailed toolset to study how rats actively and rapidly take action to confront threats in pursuit of rewards.

Humans occasionally must face threats by carrying out a voluntary action to achieve a goal. Although such an “act of courage” representing a goal-directed decision to face adverse events is critical for survival and mental health, their underlying neural circuits are barely known. Notably, a study in humans showed that the prefrontal and the temporal lobe (including the amygdala) are involved in voluntarily confronting fear of an approaching snake (Nili et al., 2010). In line with this, previous studies in humans suggest that the use of different voluntary cognitive coping strategies to inhibit fear (e.g., reappraisal) engage the same prefrontal–amygdala pathway and additional structures like the striatum and hippocampus (Hartley and Phelps, 2010). However, due to the limitations of research in humans, these studies are not able to identify specific neural circuits and mechanisms involved in this type of behavioral strategy to face threats. Ongoing studies in our laboratory, using the tasks validated here, are beginning to reveal prefrontal and subcortical (including amygdala and striatum) brain circuits necessary to choose to face threats when rewards are available during approach/avoidance conflict tests (Hernandez-Jaramillo and Sotres-Bayon, 2018; Illescas-Huerta et al., 2018). Further studies in animal models and humans are required to reveal the neural underpinnings of the ability to use goals as incentives to face adversities, which would explain an important facet of human behavior and may help treat psychiatric disorders characterized by deficits in emotional regulation.

### Clinical Implications

Combining our conflict test battery with pharmacological interventions may help find new approaches to treat mental disorders in humans characterized by deficits in decision-making in the face of conflicting emotional information. Our findings in rats suggest that a single low dose of DZPM decreases anticipatory anxiety, thereby increasing decision-making confidence to approach goals in the face of threats during conflict situations only. In humans, experiencing conflicting emotions can trigger excessive anticipatory anxiety, which is commonly accompanied by low confidence in daily decisions (Botvinick et al., 2001; Mechias et al., 2010; Goette et al., 2015; Raio et al., 2017). DZPM is one of the most widely used anxiolytic (antianxiety) drugs used by humans. Thus, our findings in rats are consistent with the notion that clinical use of an acute low dose of DZPM in humans is most effective at temporarily treating core symptoms of anxiety disorders. We suggest that such an anxiolytic effect may be specific to conflict-elicited anxiety and that it may act by increasing confidence to decide in anticipation to potentially adverse events [without altering impulsiveness (Reynolds et al., 2004)] allowing individuals to reach goals such as social competitiveness (van der Kooij et al., 2018) or psychotherapy treatment (Watanabe et al., 2007).

## Data Availability Statement

The raw data supporting the conclusions of this article will be made available by the authors, without undue reservation.

## Ethics Statement

The animal study was reviewed and approved by Institutional Animal Care and Use Committee of the Universidad Nacional Autónoma de México.

## Author Contributions

EI-H, LR-L, ROS, and FS-B: conceptualization and methodology. JAQ and FS-B: resources. FS-B: supervision and funding acquisition. EI-H, LR-L, and ROS: investigation. EI-H, LR-L, and FS-B: writing–original draft. EI-H, LR-L, ROS, JAQ, and FS-B: writing–review and editing. All authors contributed to the article and approved the submitted version.

## Conflict of Interest

The authors declare that the research was conducted in the absence of any commercial or financial relationships that could be construed as a potential conflict of interest.

## Supplementary Material

Supplementary Figure 1 A low, but not a high, dose of diazepam leaves locomotion and motricity levels intact. Two diazepam doses (1 and 2 mg/kg) were injected in rats to evaluate effects on anxiety-like behavior and locomotion in the open field test and motricity in the beam walking test. **(A)** Rats injected with either 1 mg/kg (low dose, *n* = 8) or 2 mg/kg (high dose, *n* = 9) doses of diazepam (DZPM) showed a similar number of entries to the center of the open field test than rats injected with saline solution (SAL, *n* = 17). Rats injected with a high dose of diazepam showed decreased distance traveled in the open field test, whereas rats injected with a low dose of diazepam showed similar distances traveled as compared to the saline solution group. **(B)** Rats injected with a high dose of diazepam (*n* = 8) showed increased time to arrive at the end of the walking beam, whereas rats injected with a low dose of diazepam (*n* = 6) showed similar times as compared to the saline solution group (*n* = 6). Error bars indicate SEM. ^∗^*p* < 0.05; ^∗∗^*p* < 0.01.

Supplementary Figure 2 Trial by trial examples of rats before and after injections in the crossing-mediated conflict task. **(A)** Before the injection (pre-test) saline- (SAL: Rat #1, Rat #2, and Rat#3) and diazepam-treated (DZPM: Rat #4, Rat #5, and Rat #6) rats showed similarly high crossing latencies (s) (top) and hesitation events (bottom) during the Conflict trials (black) and similarly low latencies (s) (top) and hesitation events (bottom) during the No-conflict trials (green). **(B)** Notice that after injection (Test), the diazepam-treated rats dramatically reduced crossing latencies (top) and abolished the expression of hesitation events (bottom) during conflict conditions without affecting No-conflict trials, whereas the performance of the saline-treated rats is similar to pre-test.

Supplementary Table 1 Conflict test battery. Stimuli, conditions, and apparatus used for each of the individual conflict tasks. Together these tasks constitute the behavioral test battery to study how animals confront threats to pursue rewards. In the crossing-mediated conflict task, hungry rats guided by learned aversive and appetitive cues (in a multi trial two-way straight alley), choose to seek sucrose pellets despite the threat. In the step-down mediated conflict task, thirsty rats guided by a learned aversive stimulus and an innate appetitive stimulus (in a single trial one-way chamber), choose to seek sweetened water despite the threat. In the foraging-mediated conflict task, hungry rats guided by innate aversive and appetitive stumuli (in an open field arena), choose to seek sucrose pellets despite the threat.
