# Supplementary figures and images for: Conflict Test Battery for Studying the Act of Facing Threats in Pursuit of Rewards
Source: Front Neurosci. 2021 May 4;15:645769. doi: 10.3389/fnins.2021.645769 (PMC8129192; doi:10.3389/fnins.2021.645769)

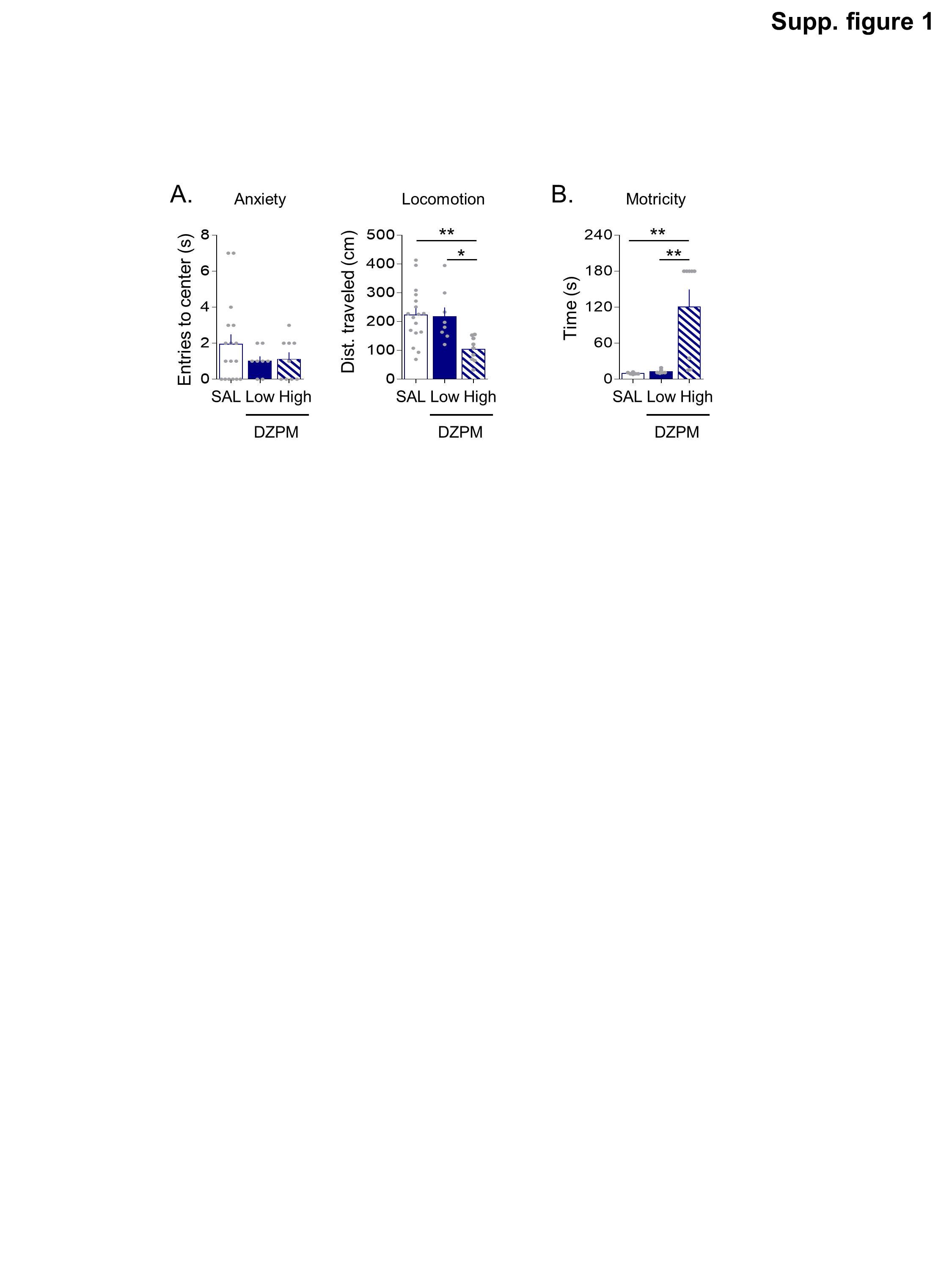

Supplement: Supplementary Figure 1 — A low, but not a high, dose of diazepam leaves locomotion and motricity levels intact. Two diazepam doses (1 and 2 mg/kg) were injected in rats to evaluate effects on anxiety-like behavior and locomotion in the open field test and motricity in the beam walking test. (A) Rats injected with either 1 mg/kg (low dose, n = 8) or 2 mg/kg (high dose, n = 9) doses of diazepam (DZPM) showed a similar number of entries to the center of the open field test than rats injected with saline solution (SAL, n = 17). Rats injected with a high dose of diazepam showed decreased distance traveled in the open field test, whereas rats injected with a low dose of diazepam showed similar distances traveled as compared to the saline solution group. (B) Rats injected with a high dose of diazepam (n = 8) showed increased time to arrive at the end of the walking beam, whereas rats injected with a low dose of diazepam (n = 6) showed similar times as compared to the saline solution group (n = 6). Error bars indicate SEM. ∗p < 0.05; ∗∗p < 0.01. [file Image_1.tif]

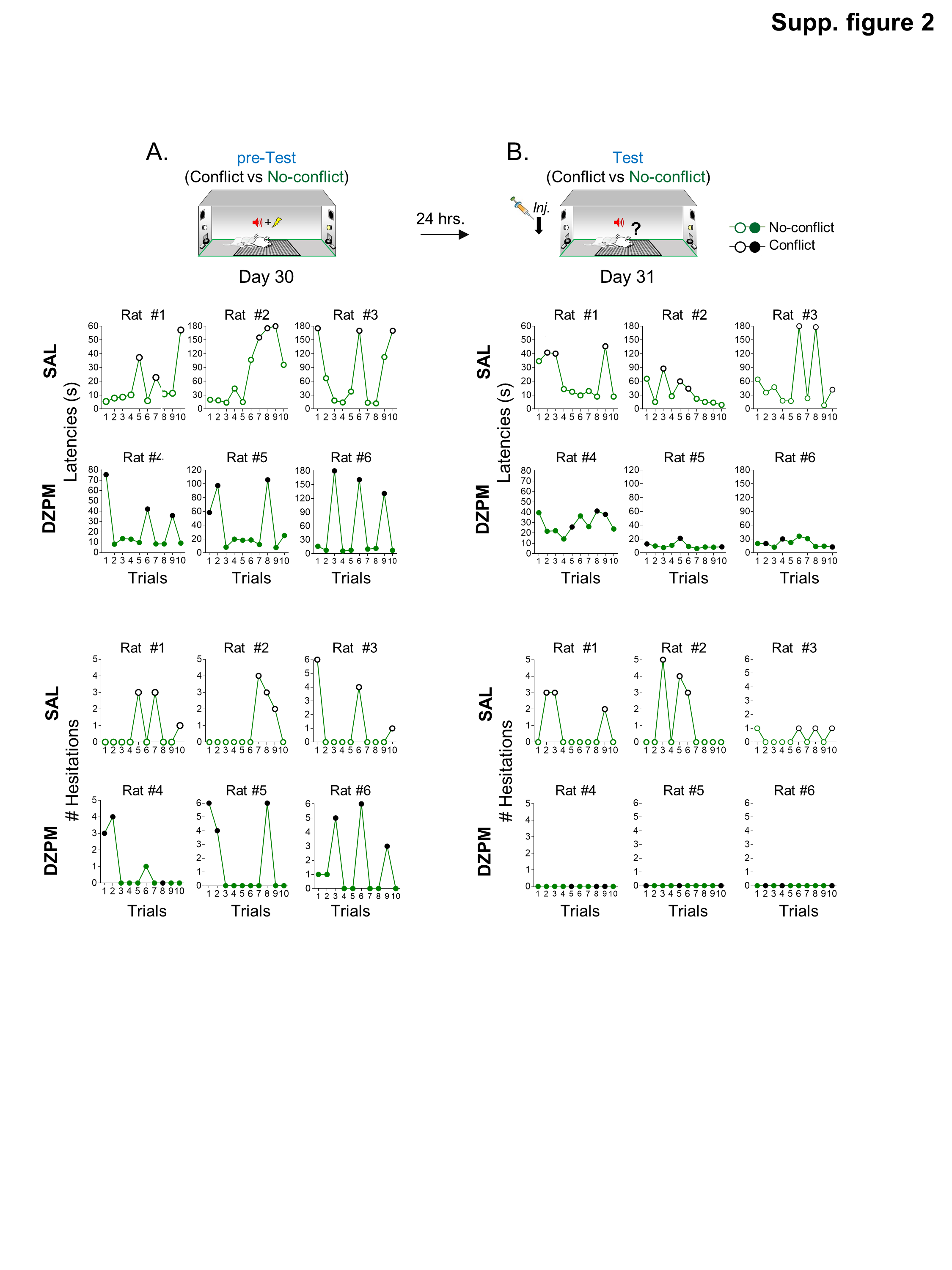

Supplement: Supplementary Figure 2 — Trial by trial examples of rats before and after injections in the crossing-mediated conflict task. (A) Before the injection (pre-test) saline- (SAL: Rat #1, Rat #2, and Rat#3) and diazepam-treated (DZPM: Rat #4, Rat #5, and Rat #6) rats showed similarly high crossing latencies (s) (top) and hesitation events (bottom) during the Conflict trials (black) and similarly low latencies (s) (top) and hesitation events (bottom) during the No-conflict trials (green). (B) Notice that after injection (Test), the diazepam-treated rats dramatically reduced crossing latencies (top) and abolished the expression of hesitation events (bottom) during conflict conditions without affecting No-conflict trials, whereas the performance of the saline-treated rats is similar to pre-test. [file Image_2.tif]

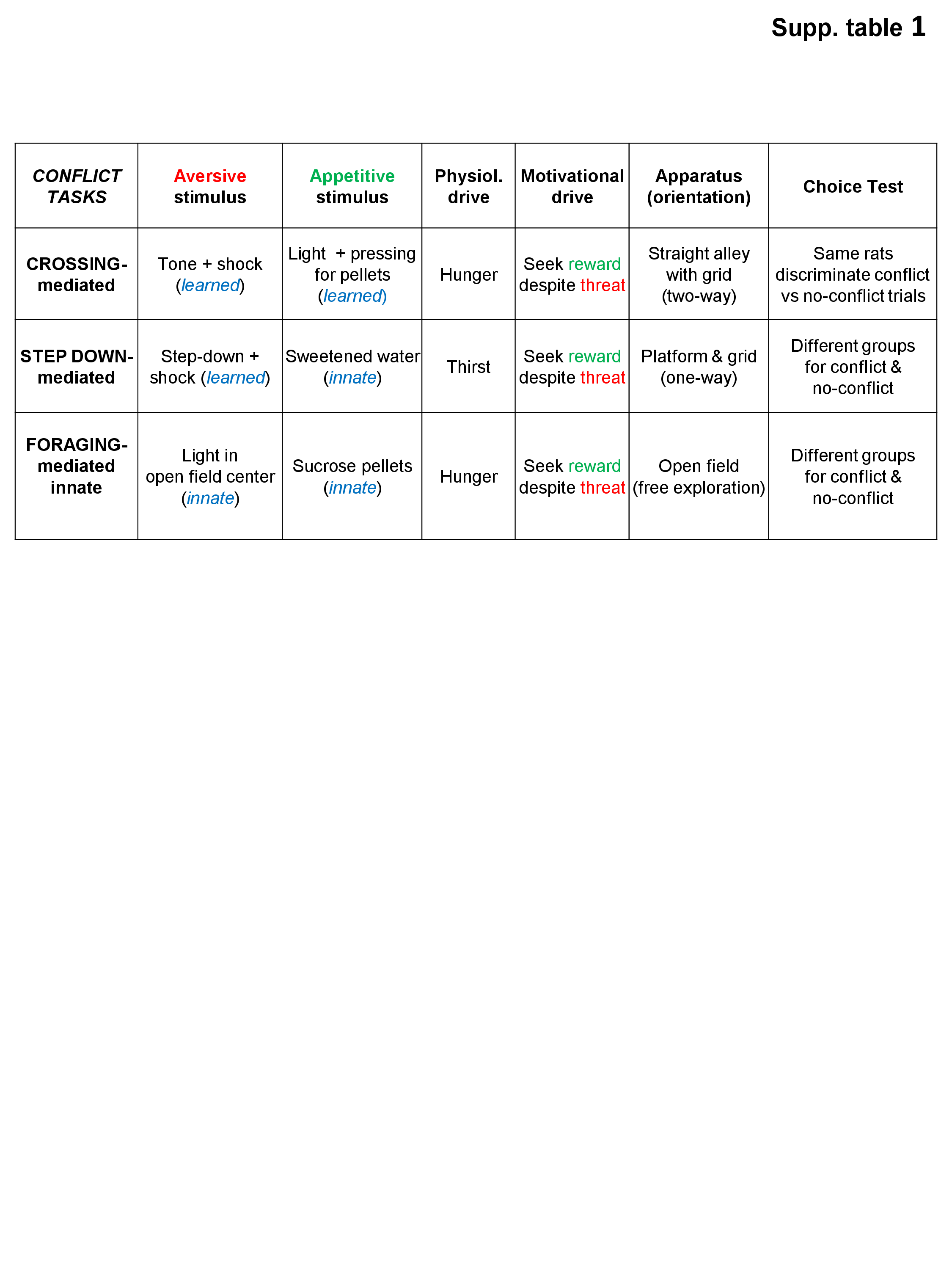

Supplement: Supplementary Table 1 — Conflict test battery. Stimuli, conditions, and apparatus used for each of the individual conflict tasks. Together these tasks constitute the behavioral test battery to study how animals confront threats to pursue rewards. In the crossing-mediated conflict task, hungry rats guided by learned aversive and appetitive cues (in a multi trial two-way straight alley), choose to seek sucrose pellets despite the threat. In the step-down mediated conflict task, thirsty rats guided by a learned aversive stimulus and an innate appetitive stimulus (in a single trial one-way chamber), choose to seek sweetened water despite the threat. In the foraging-mediated conflict task, hungry rats guided by innate aversive and appetitive stumuli (in an open field arena), choose to seek sucrose pellets despite the threat. [file Image_3.TIFF]
